# Contrasting Patterns of the Bacterial Communities in Melting Ponds and Periglacial Rivers of the Zhuxi glacier in the Tibet Plateau

**DOI:** 10.3390/microorganisms8040509

**Published:** 2020-04-02

**Authors:** Yang Hu, Xin Yao, Yuanyuan Wu, Wei Han, Yongqiang Zhou, Xiangming Tang, Keqiang Shao, Guang Gao

**Affiliations:** 1 Taihu Laboratory for Lake Ecosystem Research, State Key Laboratory of Lake Science and Environment, Nanjing Institute of Geography and Limnology, Chinese Academy of Sciences, Nanjing 210008, China; huyang@niglas.ac.cn; 2 School of Environment and Planning, Liaocheng University, Liaocheng 25200, China; 3 Sino-Japan Friendship Center for Environmental Protection, Beijing 100029, China

**Keywords:** bacterial community, melt ponds, periglacial rivers, rare sub-community, co-occurrence network

## Abstract

Since the early 21^st^ century, global climate change has been inducing rapid glacier retreat at an unprecedented rate. In this context, the melt ponds impart increasing unique footprints on the periglacial rivers due to their hydrodynamic connection. Given that bacterial communities control numerous ecosystem processes in the glacial ecosystem, exploring the fate of bacterial communities from melt ponds to periglacial rivers yields key knowledge of the biodiversity and biogeochemistry of glacial ecosystems. Here, we analyzed the bacterial community structure, diversity, and co-occurrence network to reveal the community organization in the Zhuxi glacier in the Tibet Plateau. The results showed that the bacterial communities in melt ponds were significantly lower in alpha-diversity but were significantly higher in beta-diversity than those in periglacial rivers. The rare sub-communities significantly contributed to the stability of the bacterial communities in both habitats. The co-occurrence network inferred that the mutually beneficial relationships predominated in the two networks. Nevertheless, the lower ratio of positive to negative edges in melt ponds than periglacial rivers implicated fiercer competition in the former habitat. Based on the significantly higher value of degree, betweenness, and modules, as well as shorter average path length in melt ponds, we speculated that their bacterial communities are less resilient than those of periglacial rivers.

## 1. Introduction

Glacial ecosystems, at the interface of the cryosphere, hydrosphere, pedosphere, and the biosphere, cover approximately 10% of the Earth’s surface [1] and store approximately 75% of the world’s freshwater [2]. Owing to rapid population growth, water demand exceeds supply worldwide, making glacial meltwater a crucial resource [3,4]. Thus, research to date on the practical and scientific fields has focused largely on the glacial ecosystems [5,6].

Distributed over glacial landscapes and key components of glacial ecosystems are melt ponds, which collect meltwater on the glacial surface in visible pools [7]. To date, the mechanisms of their formation and maintenance have been widely explored. Topographic depressions in the surface exert a first-order control on the formation of melt ponds, followed by a positive albedo-feedback that the lower albedo of surface waters enhances radiative-driven melting [8]. Despite the harsh and pristine environment, melt ponds are not lifeless, but support a remarkable biodiversity, including archaea, bacteria, as well as microeukaryotes [9,10,11,12]. 

The bacterial communities of melt ponds play crucial roles in influencing glacial mass balance. For instance, heterotrophic bacteria and their products reduce the surface albedo and accelerate the melting of snow and ice [13], which also deepen the melt ponds [14]. The bacterial communities in melt ponds also orchestrate nutrient and energy dynamics in glacial ecosystems [15]. For instance, previous investigations have revealed the highly production process dominated by microbial communities, such as carbon and nitrogen uptake [9,16], reductive dehalogenation [12], and even the enrichment of trace elements [17]. Thus, it is speculated that bacteria serve as the primary producers and act as the recyclers within the glacier ecosystem [18,19]. Therefore, any attempt to understand the ecology of melt ponds must be placed in the context of their bacterial communities.

Although the crucial role of bacterial communities on melt ponds has been increasingly recognized, their patterns of composition and structure are rarely studied. Alternatively, much attention has been gathered for bacterial communities in cryoconite holes, which are physically analogous to melt ponds. Phylogenetically, four major groups are represented in the communities: Proteobacteria, Actinobacteria, Bacteroides, and Cyanobacteria [13,20,21]. Wind-borne inocula and environmental variables are coupled to structure the bacterial assemblages [19,20]. However, the knowledge about the bacterial communities in melt ponds is still limited. Additionally, these aforementioned studies are limited to basic inventory descriptions at the sample level, leading to several knowledge gaps remaining, especially about interacting interactions and the role of rare taxa. Although it has long been known that bacterial species interact with each other to form complicated networks capable of community level functions, such interaction in glacial ecosystems have only been studied very recently [22,23]. Furthermore, modern ecology has recognized the over-proportional role of rare species, especially in pristine environments [24,25]. For instance, it is the rare biosphere that controls the succession strategy and ecological functions of bacterial communities during early periglacial lake ontogeny [26]. However, knowledge about the role of rare sub-communities in melt ponds is still unclear. 

On the glacier forefronts, periglacial rivers (namely glacier-fed rivers), which integrate upstream catchment processes, also constitute a prominent geomorphological and ecological component in the glacial ecosystem [27]. Owing to the close hydro-ecological coupling relationships, melt ponds profoundly impact their adjacent periglacial rivers [28]. By discharging through highly permeable ice or structural flaws, the meltwater runoff from melt ponds shapes the hydrological regimes, sediment transport, biogeochemical, and contaminant fluxes of periglacial rivers [29]. 

In the last 30 years, atmospheric warming has been aggravated, causing climate-induced glacier retreat. This in turn changes the influences of melt ponds on their periglacial rivers in terms of speciation of nutrient, hierarchical habitat template, and ecosystem services [1,27,30]. However, how the bacterial communities change from melt ponds to periglacial rivers is still unknown. The organisms in melt ponds are delivered downstream, facilitating the displacement of less cryophilic species by cold-adapted species in the periglacial rivers [27]. While melt ponds offer a relatively constant environment for bacterial life, periglacial rivers are highly dynamic and, thus, organisms are exposed to massive temporal fluctuations. Water temperature, discharge, channel stability, and sediment transport can significantly fluctuate for bacterial life [12], which also have major implications for the biogeochemistry of downstream ecosystems. In this regard, the worldwide glacier recede creates an urgent need to evaluate the fate of bacterial communities from melt ponds to periglacial rivers.

To uniquely characterize the glacial bacterial communities in glacial ecosystems during deglaciation succession, we comprehensively characterized and compared the bacterial communities from melt ponds to periglacial rivers, regarding their structure, diversity, and co-occurrence network. This investigation sheds new insight on the ecological succession for bacterial communities under the global warming scenario. Specifically, we hypothesized that: (1) the rare sub-community contributes to the bacterial community in the glacial ecosystem; and (2) the bacterial communities and their co-occurrence networks are distinct between melt ponds and their periglacial rivers.

## 2. Materials and Methods

### 2.1. Study Area and Sampling

**Figure 1:**
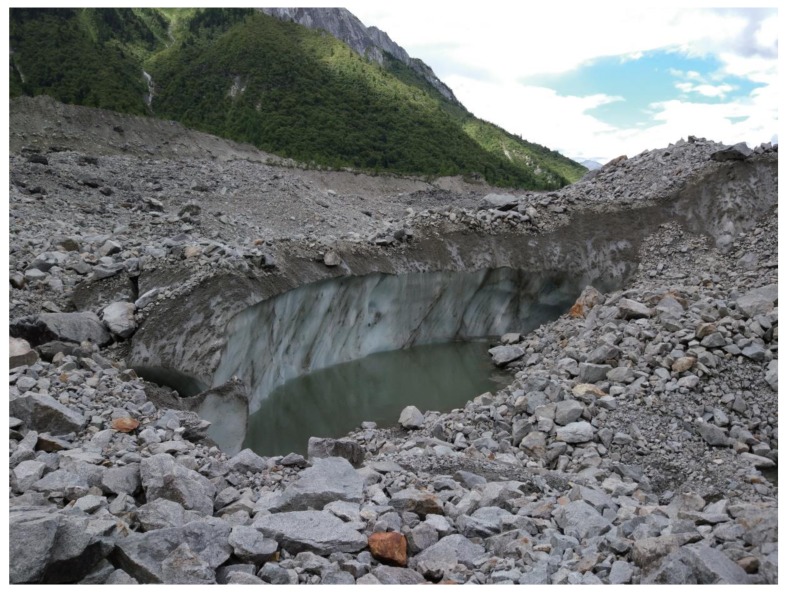

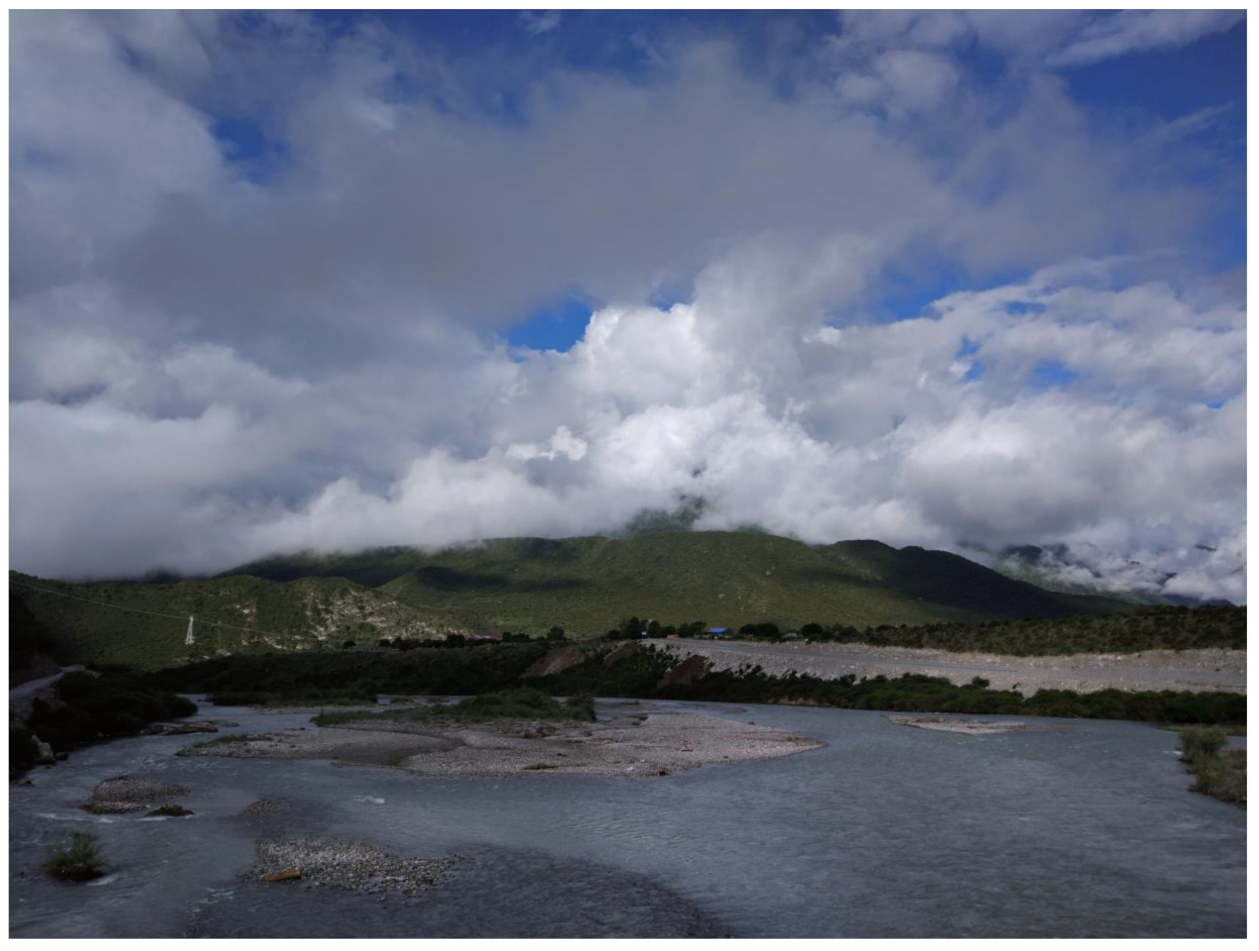
General view of melt ponds (top) and periglacial rivers (bottom) in the Zhuxi Glacier.

Our field work was carried out on the Zhuxi Glacier (30°01′N, 95°30′E), which lies at an elevation of 3500 m on the Tibetan Plateau in October 2017 (Figure 1). The India summer monsoon enters the Zhuxi Glacier from the southeast, bringing moisture from the Indian Ocean during the summer. Based on meteorological records, the mean annual precipitation in the study area is 837 mm. July (mean temperature of 15.8 °C) and January (mean temperature of 0.5 °C) are the warmest and coldest months, respectively [31]. Melt ponds in this region are half or completely open. For our research, we chose nine melt ponds at approximately 500 m intervals, each with a diameter of approximately 100 cm and a depth of approximately 50 cm. Each of the ponds flowed directly into periglacial rivers. We also studied 13 periglacial rivers which were some 1000 m downstream of the glacier forefront.

Water samples (mixed top, middle, and bottom) were collected with a 5 L Schindler sampler, then were placed in sterile polyethylene bottles, and were stored in a frozen condition. A 1500 mL subsample for 16S rRNA gene analysis was filtered onto a 0.2 μm polycarbonate membrane (47 mm diameter, Millipore, Billerica, MA, USA) under the vacuum pressure of < 15 mm Hg. 

*In situ* measurements of water temperature (Temp), turbidity (Turb), and dissolved oxygen (DO) were measured by a multi-parameter water quality sonde (YSI 6600v2; USA). Total nitrogen (TN), dissolved total nitrogen (DTN), total phosphorus (TP), and dissolved total phosphorus (DTP) were determined, according to the standard methods [32], using a combined persulfate digestion; the NH_4_ was determined using the indophenol blue method and NO_3_ with the cadmium reduction method. The dissolved organic carbon (DOC) was determined using a total organic carbon analyzer (TOC-VCPH; Shimadzu Scientific Instruments, Columbia, MD, USA).

### 2.2. DNA Extraction, PCR, and Illumina Miseq Sequencing

Crude DNA extracts were purified by the E.Z.N.A^®^ cycle-Pure kit (Omega Bio-Tek, Norcross, GA, USA). The V4 region of the 16S rRNA genes were amplified using the primers 515F (GTGYCAGCMGCCGCGGTAA) and 806R (GGACTACNVGGGTWTCTAAT) [33]. Polymerase chain reaction (PCR) amplification was performed in a 50 μL reaction mixture, containing 5 μL of 10 × PCR buffer, 4 μL of MgCl_2_ (25 mmol L^−1^), 0.5 μL of each primer (10 μmol L^−1^ each), 30 ng quantified template DNA measuring by Pico green, and 0.4 μL of *Taq* polymerase (5 U μL^−1^; MBI Fermentas, Hanover, MD, USA). The PCR cycling was conducted in a thermocycler (Applied Biosystems Veriti Thermal Cycler, Thermo Fisher Scientific, MA, USA) by a touchdown program: denaturation at 94 °C for 5 min, 11 cycles of denaturation at 94 °C for 1 min, annealing at 65 °C for 1 min (temperature was decreased by 1 °C every cycle until 55 °C was reached), and extension at 72 °C for 1 min. Nineteen additional cycles were performed at an annealing temperature of 55 °C, followed by a final extension at 72 °C for 10 min.

Clean amplicon pools for 22 samples were paired-end sequenced (2 × 250) on an Illumina Miseq platform at Magigen Biotechnology (Guangzhou, China). We removed sequences if they: (i) contained more than one ambiguous nucleotide, (ii) lacked a complete barcode and primer at one end, and (iii) were shorter than 200 bp after removal of the barcode and primer. Sequences were merged and denoised with USEARCH (version 10.0.240) [34] and then were clustered with 97% similarity cutoff into unique sequences (operational taxonomic units; OTUs) using the UNOISE. Chimeras were identified and removed with UCHIME and subsequently also removed with a reference-based comparison against SSU SILVA NR database (release 132, www.arb-silva.de). The representative sequence of each phylotype was selected and aligned by RDP Classifier (version 16) against the SILVA 16s rRNA database (release 132) with a confidence threshold of 80%. We excluded from downstream analyses low confidence singletons that had a sequence count smaller than 2.

### 2.3. Network Construction and Characterization

We constructed two individual co-occurrence networks of the bacterial communities for melt ponds and periglacial rivers using the CoNet (v.1.1.1.beta) plugin in Cytoscape (v3.5.1), as previously described [35]. We included only OTUs that appeared in all 22 samples [36]. In each network, pair-wise associations among OTUs were simultaneously identified by an ensemble of correlations (Pearson and Spearman coefficients) and distance metrics (Kendall distance, Bray-Curtis distance, and Kullback-Leibler dissimilarity measures). The initial 500 top- and bottom-ranking edges were kept in the network. A total of 1000 permutation scores and 1000 bootstrap scores were calculated for each edge and each measure of association [37]. The measure-specific *p*-values from multiple interaction metrics were merged using the Simes method [38] and a false-discovery rate correlation was performed using the Benjamini-Hochberg multiple testing correction [39]. 

The network was explored by the “*igraph*” package [40]. To describe the network topology, a set of properties was calculated, including number of nodes and edges, transitivity, betweenness, average path length, and modularity. To isolate modules, we applied the algorithm of fast greedy modularity optimization [41]. The keystone nodes were identified though GIANT plugin in the Cytoscape [42].

### 2.4. Statistical Analysis

All analyses and visualizations were prepared by *vegan* [43] and *ggplot2* [44] in the R environment (version 3.2.2, http://www.r-project.org). Data sets of multivariate statistical analyses, diversity estimates, and co-occurrence network construction were rarefied according to the lowest numbers of reads among all samples. Previous studies have claimed that abundant phylotypes are defined as having relative abundance >0.1% at the local level (one sample) [45]. However, this value was inappropriate in our study because there were few “abundant” OTUs according to this definition. Thus, we differentiated between abundant and rare members using a threshold of 0.01% at the regional level (across samples, abundant members ≥0.01%, rare members < 0.01%) [23]. To determine the contribution of rare sub-community to the variation of bacterial community, rare OTUs were stepwise included based on the Raup-Crick metric, which allows comparison of the beta-diversity independent of changes in alpha-diversity [26,46]. As the quantification of variation of bacterial community, we measured the mean distance among all samples in the ordinations. This approach creates a re-scaled probability value ranging from −1 to 1, indicating whether the bacterial communities are more dissimilar (approaching 1), as dissimilar (approaching 0), or less dissimilar (approaching −1) than expected by stochastic (null) expectations.

We determined four measures of the alpha-diversity of the bacterial community: Chao1, Faith’s phylogenetic diversity (Faith’s PD), Shannon index, and Simpson index. Faith’s PD was based on the phylogenetic tree built with representative OTUs by using FastTree (version 2.1.10). The alpha-diversity and environmental properties between melt ponds and periglacial rivers were compared by Kruskal-Wallis test through the *kruskal.test* function. To compare the relative abundance of bacterial taxa, STAMP (version 2.0.9) was used. To determine the beta-diversity of the bacterial community, we conducted non-metric multidimensional scaling (NMDS) using the *metaMDS* function based on the four distance matrices: Bray-Curtis distance, Jaccard dissimilarity, and (un)Weighted UniFrac distance. The variations in bacterial communities between habitats were compared by *betadisper* function. The statistical differences between bacterial community composition were determined by the PERMANOVA test using the *adonis* function. 

## 3. Results

### 3.1. The Physicochemical Conditions in Melt Ponds and Periglacial Rivers

**Figure 2:**
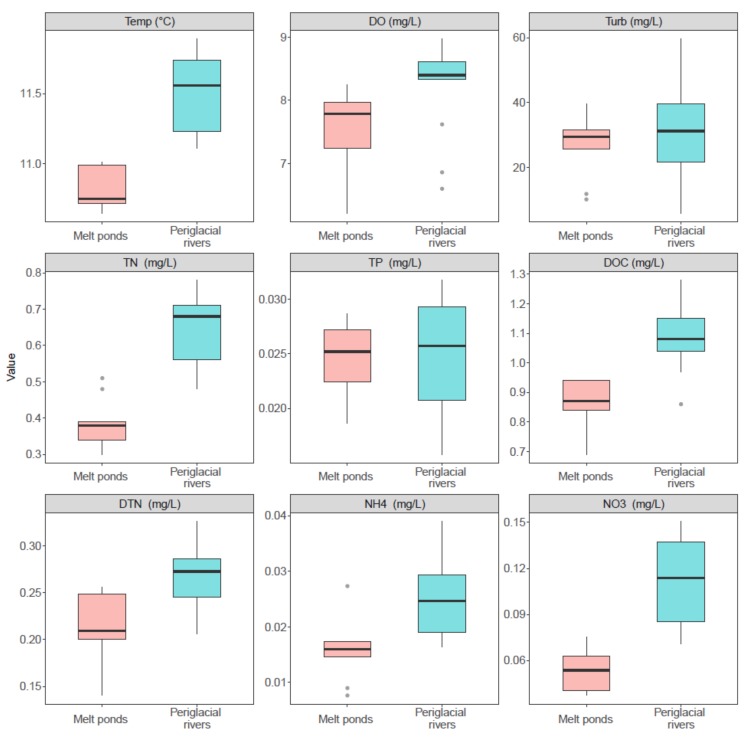
Comparison of aquatic characteristics between melt ponds and periglacial rivers.

Our results showed that the aquatic environments were inherently distinct between melt ponds and periglacial rivers (Figure 2). The water temperature and DO significantly increased from melt ponds to periglacial rivers (Kruskal-Wallis test, *p* = 0.01 and *p* < 0.001, respectively). Furthermore, the concentrations of nutrients, including TN, NH4, NO3, DTN, and DOC, were significantly higher in periglacial rivers than in melt ponds (all *p* < 0.001). In contrast, TP was comparable between two habitats (*p* = 0.92). Turbidity increased from 26.37 ± 13.53 NTU to 31.20 ± 9.00 NTU, but with no significance (*p =* 0.44). DTP was below the detection limitation in both habitats.

### 3.2. Diversity of Bacterial Communities between Melt Ponds and Periglacial Rivers

After quality filtering and removing chimeras, the Illumina sequencing yielded an average of 88,767 high-quality sequences with 235 bp for each site. The Good’s coverage was 98.69%–99.78%, suggesting that the sequencing effort captured most of the bacterial diversity. This implication was also supported by the rarefaction curves, which approached the saturation plateau in all 22 communities (Figure S1). The reserved sequences were clustered into 5892 OTUs across all sampling sites. Specifically, 3894 ± 640 OTUs and 5078 ± 225 OTUs were classified to melt ponds and periglacial rivers, respectively. A total of 32.36% of OTUs were detected in all 9 samples from melt ponds, while 55.10% of OTUs were detected in all 13 samples from periglacial rivers. For melt ponds, 93.22% of the total OTUs belonged to the rare sub-communities, which accounted for 64.82% of the whole reads. For periglacial rivers, the rare sub-communities consisted of 91.84% of the total OTUs, which accounted for 54.26% of the whole reads. 

**Figure 3:**
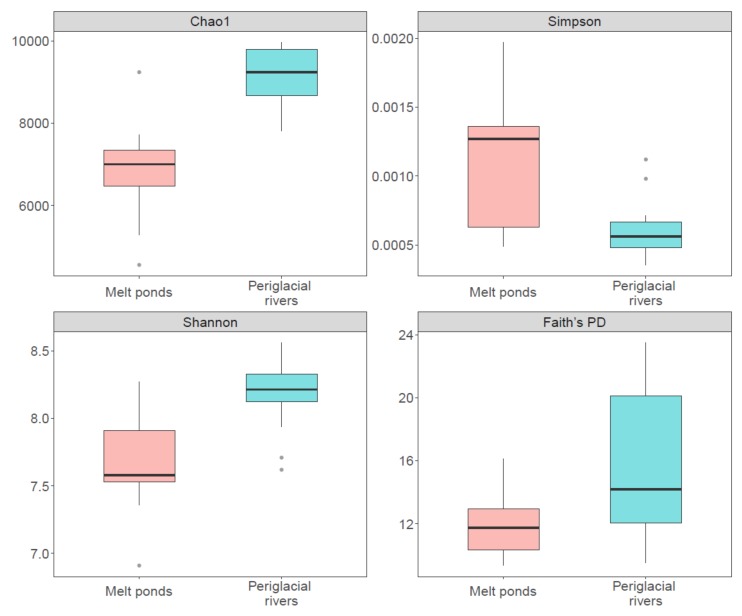
The alpha-diversity of bacterial communities in melt ponds and periglacial rivers.

**Figure 4:**
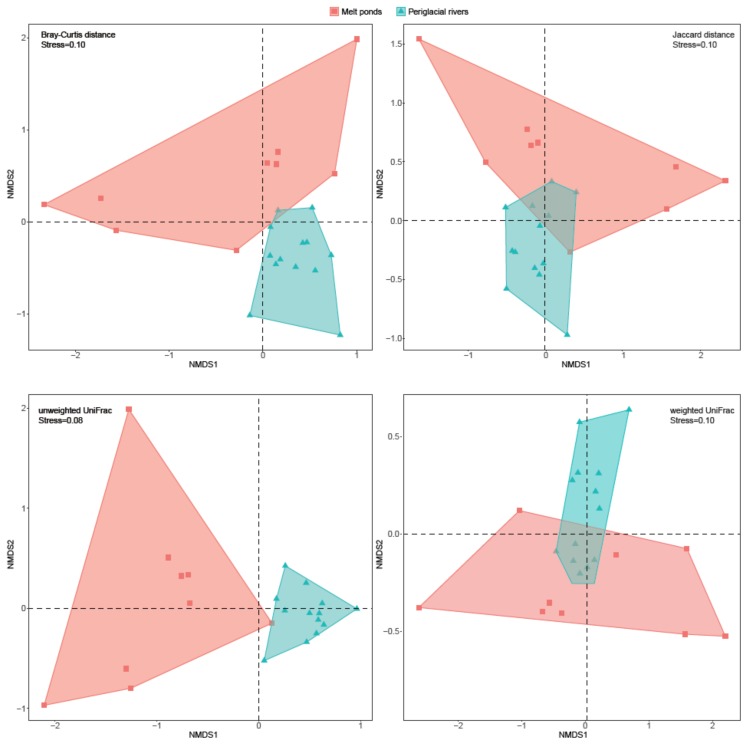
Non-metric multidimensional scaling (NMDS) plots of bacterial communities in melt ponds and periglacial rivers based on four distance matrices.

**Figure 5:**
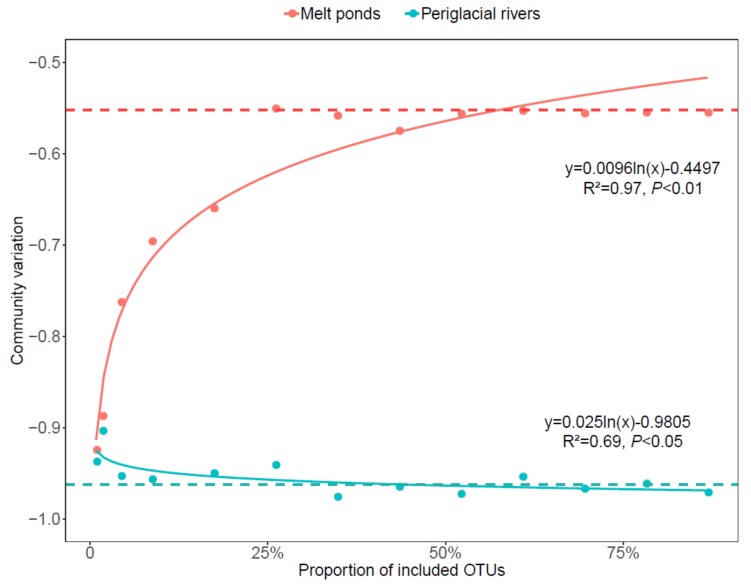
The bacterial community variation (based on Raup-Crick metrics) along a sequence of accumulating rare OTUs in melt ponds and periglacial rivers.

All four indices of community alpha-diversity showed that that the bacterial communities in melt ponds were significantly less diverse than those in periglacial rivers (*p* < 0.001, *p* = 0.02, *p* < 0.001, and *p* = 0.04 for Chao1, Simpson, Shannon, and Faith’s PD, respectively) (Figure 3). Within both habitats, the rare sub-communities showed significantly higher alpha-diversity than the abundant sub-communities (Figure S2, *p* = 0.001 and *p* = 0.002 for melt ponds and periglacial rivers, respectively). The beta-diversity pattern of the bacterial communities in melt ponds and periglacial rivers were also evaluated. The NMDS plot showed a clear segregation between the bacterial communities in the two habitats (Figure 4), which was confirmed by the PERMANOVA test (*p* = 0.001). Based on the four distance matrixes, the permuted analysis of betadispersion of pairwise similarities showed that the bacterial communities in melt ponds were significantly more variable than those in periglacial rivers (*p* < 0.001). Furthermore, to explore the influence of the rare sub-communities on their whole communities, we calculated the bacterial community variation along a sequence of an accumulation of rare OTUs (Figure 5). The stepwise inclusion of rare OTUs leaded significantly to an asymptote of community variation in both melt ponds and periglacial rivers, which gradually paralleled that of the whole bacterial communities.

### 3.3. The Taxonomic Profile of the Bacterial Communities in Melt Ponds and Periglacial Rivers

We compared the taxonomic structure of bacterial communities in melt ponds and periglacial rivers (Figure S3). The sequences were classified and grouped under 9 phyla in both melt ponds and periglacial rivers, which were dominated by Proteobacteria (47.79% versus 57.48%) and Bacteroidetes (32.11% versus 26.72%). The STAMP analyses showed that only Proteobacteria and Verrucomicrobia were significantly different between melt ponds and periglacial rivers, with both being significantly higher in the latter habitat (both *p* < 0.001, Figure S4).

At a finer taxonomic level, 17 and 18 classes were detected in melt ponds and periglacial rivers, respectively. In melt ponds, the dominant class was Gammaproteobacteria (20.33%), followed by Betaproteobacteria (18.12%), Sphingobacteriia (14.06%), and Bacilli (15.31%). In contrast, in periglacial rivers, the dominant class was Alphaproteobacteria (26.65%), followed by Gammaproteobacteria (19.81%), Flavobacteriia (14.45%), and Bacilli (12.54%). STAMP analysis indicated that only three classes were significantly different between the two habitats, with Betaproteobacteria being significantly higher in melt ponds (*p* = 0.04), while Alphaproteobacteria, Spartobacteia, and Verrucomicrobiae were significantly higher in periglacial rivers (both *p* < 0.001, Figure S5). 

At the genus level, the sequences from melt ponds and periglacial rivers represented 83 identified genera. In melt ponds, the most abundant genus (average abundance > 5%) was *Exiguobacterium* (14.56%), followed by *Flavobacterium* (11.26%), *Ferruginibacter* (8.86%), and *Polaromonas* (7.62%). In periglacial rivers, the most abundant genera were dominated by *Flavobacterium* (14.45%), *Sphingorhabdus* (14.18%), Exiguobacterium (11.95%), and *Acinetobacter* (5.75%). STAMP analysis showed that 10 of the 83 genera significantly differed between the two habitats. *Polaromonas*, *Rhizobacter*, *Sediminibacterium*, and *Variovorax* were higher in melt ponds (*p* = 0.04, 0.03, 0.03, and 0.03, respectively, Figure S6), whereas *Sphingorhabdus*, *Novosphingobium*, *Luteolibacter*, *Sphingomonas*, *Lacihabitans,* and *Rhodopirellula* were higher in periglacial rivers than in melt ponds (all *p* < 0.001, Figure S6). 

### 3.4. Architecture of Bacterial Co-Occurrence Network between Melt Ponds and Periglacial Rivers

**Figure 6:**
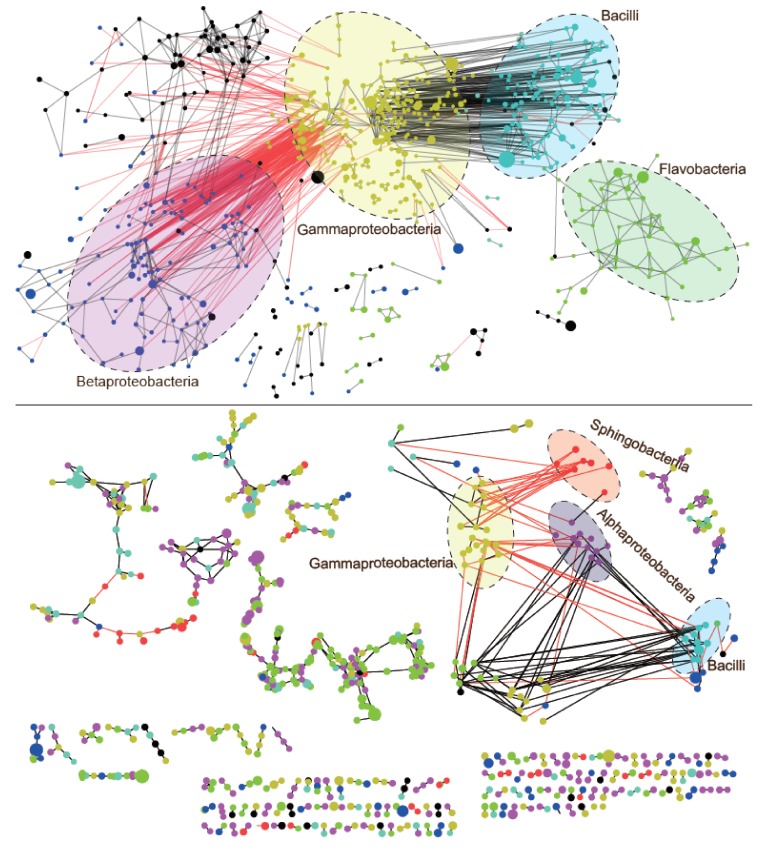
Co-occurrence networks of bacterial communities in melt ponds (top panel) and periglacial rivers (bottom panel). The red lines represent negative interactions while black lines represent positive interactions. The coloured nodes are labeled with their class information, while the black nodes without any taxonomic annotation belong to Others and Unclassified OTUs.

**Figure 7:**
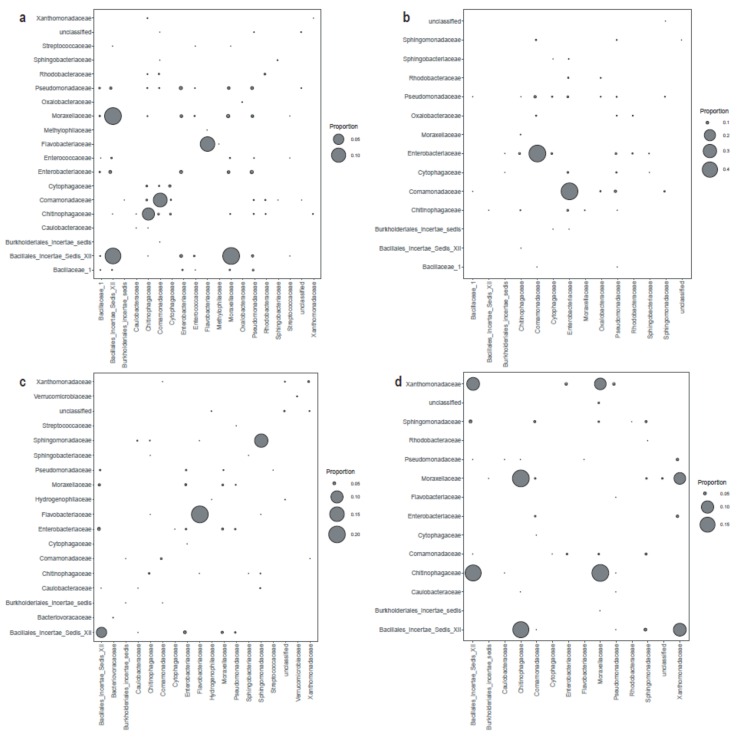
The pairwise taxonomic structure of the network edges in melt ponds and periglacial rivers. (**a**): the positive edges in melt ponds, (**b**): the positive edges in melt ponds, (**c**): the positive edges in periglacial rivers, (**d**): the positive edges in periglacial rivers. The size of the bubble represents the proportion of edges in the co-occurrence network.

The co-occurrence network of bacterial communities was more complex in melt ponds than in periglacial rivers (Figure 6). For melt ponds, the network consisted of 704 nodes which were connected by 1507 edges, while the network of the periglacial rivers contained 737 nodes and 860 edges. Remarkably, the nodes in both networks were principally composed of rare OTUs (679 and 722, respectively). In addition, the networks in melt ponds and periglacial rivers had 1159 and 660 positive edges, respectively, but only 348 and 94 negative edges, respectively; the ratio of positive to negative edges clearly was 3.33 for melt ponds and 7.02 for periglacial rivers. The taxonomic compositions of the pairwise interactions were also investigated (Figure 7). For the positive edges, the largest bubbles were located at the diagonal line, suggesting that the beneficial interactions were mainly intra-taxon interactions. In contrast, for the negative edges, the largest bubbles were located on the upper and lower areas, indicating that the mutually exclusive interactions were mainly inter-taxon interactions.

The black dashed line represents the variation of whole bacterial communities of melt ponds and periglacial rivers.

The network inference showed that the network of melt ponds had significantly higher degree (4.28 versus 2.33, *p* = 0.002), higher levels of betweenness (1187.19 versus 52.75, *p* < 0.001), and shorter average path length (3.29 versus 4.84 *p* = 0.03) than that of periglacial rivers. Additionally, the network of melt ponds was divided into less modules (64) than that of periglacial rivers (148) (*p* = 0.001). We also explored the keystone nodes by GIANT plugin in the Cytoscape. The results identified 7 and 12 keystones for melt ponds and periglacial rivers, respectively. For the melt ponds, the keystones were affiliated with *Enterobacteriaceae* (five nodes) and *Polaromonas* (two nodes). In contrast, the keystone nodes in the periglacial rivers were more phylogenetically diverse, including *Acinetobacter* (one node), *Exiguobacteriummexicanum* (five nodes), *Flavobacterium* (two nodes), *Terrimonas* (two nodes), and *Xanthomonadaceae* (two nodes). 

Notably, all seven keystone nodes in melt ponds and 11 of 12 keystone nodes in the periglacial rivers were affiliated with the rare OTUs. To evaluate the influence of the rare nodes on the community network, we applied “a brute-force leave-out strategy”. We compared the co-occurrence networks with the removal of rare keystone nodes versus removal of abundant nodes. The results showed that the removal of rare nodes significantly reduced the network size and degree. In contrast, the removal of abundant nodes has no significant effect on network size or degree.

## 4. Discussion

### 4.1. The Diversity and Composition Patterns between Melt Ponds and Periglacial Rivers

We determined that at our study site in the Zhuxi Glacier in the Tibet Plateau, the alpha-diversity of bacterial communities was significantly lower in melt ponds than in periglacial rivers. This finding is in agreement with previous studies of glacier-fed rivers, where the diversity of benthic biofilms [27,29] and macroinvertebrates [47,48] increased with increasing distance from the glacial terminus. We propose two possible mechanisms for this phenomenon. First, the colonization of bacterial communities in melt ponds is mainly derived from the deposition of wind- and water-borne materials. In contrast, the size of metacommunity in periglacial rivers is larger, which collects bacterial communities from various glacial and non-glacial sources, thereby achieving the higher alpha-diversity. The second reason may lie in the greater harshness of the abiotic environment and more limited resource availability in melt ponds compared to periglacial rivers. We showed that the concentration of carbon and nitrogen significantly increased from melt ponds to periglacial rivers. Additionally, recent evidence has also confirmed that dissolved organic materials are more bioavailable in the glacier-fed rivers than in melt ponds [49]. Thus, periglacial rivers are expected to harbor more niches for bacterial communities than are melt ponds. 

For melt ponds and periglacial rivers, the bacterial communities were dominated by Proteobacteria, Bacteroidetes, and Firmicutes, which accounted for more than 95% of the whole communities. However, several bacterial phylotypes at the finer levels significantly varied between these two habitats. For instance, the closely related genera *Polaromonas* and *Variovorax*, belonging to the family Comamonadaceae of the class Betaproteobacteria, were significantly higher in melt ponds. They are identified as psychrotolerant bacteria in glacier, ice, or snow habitats, i.e., Antarctica, Artic, the Himalayas, and the Alps [50,51]. The complete genome sequencing has revealed their unusual catabolic traits in extremely cold environments [52]. By contrast, the genera *Sphingorhabdus*, *Novosphingobium*, and *Sphingomonas*, belonging to the family Sphingomonadaceae of the class Alphaproteobacteria, were significantly higher in periglacial rivers. These bacterial phylotypes are obligate aerobic organisms that have been obtained from a wide variety of environments, including temperate and polar soil as well as sediments [53,54]. Additionally, they have also been isolated from plant tissues as the common endophytes [55]. Thus, their prevalence in periglacial rivers indirectly implies the bacterial colonization from river shore and bed. Together, these habitats-specific bacterial groups could reflect the distinct environment characteristics for melt ponds and periglacial rivers.

Remarkably, previous studies have documented that Cyanobacteria commonly dominate in glacial habitats, such as Arctic and Antarctic (e.g., Svalbard Glacier, Lower Wright Glaciers, and Diamond Glacier) and other alpine regions (Tibetan Plateau and high-mountain Asia) [13,19,20,22]. In such sites, the Cyanobacteria accounted for 20% to 40% of the whole bacterial assemblages. Strikingly, Cyanobacteria were not detected in any of our 22 sampling sites. In our present study, turbidity ranged from 10.27 to 59.54 NTU in melt ponds and their fed rivers, implicating a high concentration of suspended solids. These particles (so-called glacial flour or glacial milk) may result in the absence of Cyanobacteria. Cyanobacteria are typically photoautotrophs which serve as important primary producers in aquatic ecosystems. However, the suspended solids decrease the depth of the euphotic zone and decrease the intensity of underwater illumination [26]; both circumstances jeopardize the growth and survival of Cyanobacteria [56]. The negative effect of high levels of suspended minerals on other aquatic biota (including *Daphnia*, heterotrophic nanoflagellate (HNF), and fish species [10,11]) has been observed in glacial habitats.

### 4.2. The Role of Rare Sub-Communities on the Bacterial Communities in Melt Ponds and Periglacial Rivers

Bacterial communities commonly show a skewed species abundance distribution with only a few dominant phylotypes coexisting along with an extremely high number of rare species. This pattern is in line with our results that in each habitat, more than 90% of the total OTUs made up the rare sub-communities, which accounted for 64.82% and 54.26% of the whole reads for melt ponds and periglacial rivers, respectively. Additionally, the stepwise inclusion of rare OTUs significantly stabilized the bacterial communities in melt ponds and periglacial rivers, implying that the rare biosphere is crucial for these bacterial assemblages. Recent advances in molecular biology and cultivation techniques provide an increasing body of evidence that potentially supports this implication. For instance, Peter *et al*. demonstrated that the rare biosphere even determined the succession strategy of the bacterial communities during early ontogeny of meltwater-lakes [26]. Jiang *et al*. also reported that the rare microbiome exhibited sensitive responses to glacier retreat in Hailuogou Glacier in the Tibetan Plateau [23]. 

An important trait of the rare biosphere is the extremely high diversity, which facilitates their contributions to the entire communities [57] and has been termed the “seed bank” effect [24,25]. Within melt ponds and periglacial rivers, rare sub-communities were significantly more diverse than the dominant sub-communities. As has been demonstrated, rare sub-communities generally serve as a genetic resources pool, offering a reservoir of biodiversity for the bacterial community. Under favorable conditions, the members of rare sub-communities would leave the “seed bank” and enter the abundant sub-communities [24]. This ecological process may be inferred from the contrary size trends of these two sub-communities from melt ponds to periglacial rivers, namely that the rare sub-communities decreased while the dominant sub-communities increased. 

In our present study, co-occurrence networks in melt ponds and periglacial rivers suggested that most of the involved nodes belonged to the rare sub-communities. This pattern has previously been reported from other glacial environments in Tibet Plateau [23] and from other habitats, such as soil [58,59], rivers [60], and oceans [61]. Moreover, the over-proportional roles of rare taxa were also shown by our finding that all 7 keystones in melt ponds and 11 out of 12 keystones in periglacial rivers were affiliated with rare OTUs. As previous studies implied, the rare biosphere represented an underestimated backbone of bacterial communities in glacial ecosystems [24]. Support for this came from our computational simulation, which revealed that the removal of the rare OTUs significantly jeopardized the interspecific interactions, which may result in loss of community network stability [17]. Similarly, recent experimental evidence also showed that the removal of rare species facilitates the alien invasion and subsequently reduces community robustness [16]. Given these facts, the rare sub-communities are structurally capable of holding together populations and organizing community interaction. 

### 4.3. Higher Competitive and Connected Network of Bacterial Communities in Melt Ponds than in Periglacial Rivers

Bacteria live in a complex network through various types of interactions, which can be categorized into mutually beneficial and exclusive interactions. Within melt ponds and periglacial rivers, network inference indicated that positive correlations prevailed in the bacterial communities. This correlative pattern is in line with that reported from other habitats, including soil [62], rivers [60], and other glacial environments [26,27]. As previously suggested [37], positive association in a network is interpreted as cross-feeding, co-colonization, and co-aggregation. Together, these observations highlight a self-structured and self-sustaining assortment of bacterial communities [62]. Further, we found that most of the positive interaction was inter-taxon pairs. This result implies that phylogenetically close species tend to have strong mutualistic interactions [63], which may be related to their similar niche preferences and synergetic relationships. However, despite the predominance of positive correlations, melt ponds had a lower ratio of positive to negative correlations than did the periglacial rivers (3.33 versus 7.02, respectively). Combined with the lower concentrations of aquatic nutrients in melt ponds, the bacterial taxa are expected to compete more fiercely in melt ponds than in periglacial rivers. Unlike the positive correlation associated with inter-taxa interaction, most of the negative correlations were intra-taxa pairs. However, due to the false positive rate and apparent sensitivity to settings of the co-occurrence network [64], current results have limited conclusiveness and should be interpreted cautiously. To verify these inferred conclusions, more sophisticated forms of analyses and even laboratory evidence are required. 

With the edges only predicting biotic interactions, other emergent properties are more reliable, such as degree, betweenness centrality, modularity, and average path length [64]. The network inference showed that melt ponds exhibited higher degree and betweenness than the periglacial rivers, suggesting that bacterial taxa are more connected in melt ponds than in periglacial rivers. Moreover, the bacterial communities of melt ponds were significantly divided into less modules, which are biologically interpreted as functional units [65], divergent selection, as well as niches [66,67]. We ascribe their distinct topologies to the environmental heterogeneity between these two habitats. Based on the increasing nutrient resource from melt ponds to periglacial rivers, a niche difference is expected, which allows the bacterial communities to adapt quickly to available nutrients without the need to establish interactions with neighboring microorganisms [68]. As a consequence, the higher connectivity of bacterial communities in melt ponds would result in greater vulnerability to disturbance because the whole community will be more affected by the other nodes [58]. In contrast, we found that the network in melt ponds had significantly shorter average path length than did that of the periglacial rivers. As one of the important properties, network path is biologically viewed as a robust flow of nutrients and energy [64]. Short path length will facilitate efficient and rapid communication among different members and will allow the local perturbations to reach the community network quickly so that they may be less resilient to environmental changes. Together, the distinct network topologies between melt ponds and periglacial rivers imply that the bacteria of melt pond communities would be more variable than those in periglacial rivers. This is the case for our permuted analysis of betadispersion as beta-diversity of bacterial communities in melt ponds was significantly higher. 

## 5. Conclusions

The bacterial communities are significantly different between melt ponds and periglacial rivers in the Zhuxi Glacier on the Tibet Plateau. Specifically, owing to the pristine condition and smaller size of metacommunities, the bacterial communities in melt ponds are less diverse than those of periglacial rivers. In contrast, the bacterial communities of melt ponds are more variable across all sampling sites than are those of periglacial rivers. The co-occurrence network infers that the positive correlation is mainly the intra-taxon pair, while the negative correlation is mainly the inter-taxon pair, and that the rare sub-community significantly stabilized the whole bacterial community in both habitats. Moreover, the bacterial communities in melt ponds may be more competitive and more connected than those in periglacial rivers. In short, these observations suggest the bacterial communities of melt ponds may be more sensitive to environmental change than those in periglacial rivers. This study provides a substantial framework to guide the study of micro-ecology in glacier ecosystems.

## Supplementary Materials

The following are available online at https://www.mdpi.com/2076-2607/8/4/509/s1, Figure S1. The rarefaction curves of the bacterial communities in melt ponds and periglacial rivers. MP represents melt ponds and PR represents periglacial rivers. Figure S2. The alpha-diversity of dominant and rare sub-communities in melt ponds and periglacial rivers. Figure S3. Community composition of the bacterial communities in melt ponds and periglacial rivers at the phylum, class, and genus levels. Figure S4. The STAMP analysis of the bacterial communities at the phylum level between melt ponds and periglacial rivers. Figure S5. The STAMP analysis of the bacterial communities at the class level between melt ponds and periglacial rivers. Figure S6. The STAMP analysis of the bacterial communities at the genus level between melt ponds and periglacial rivers.
