# Supplementary material for: Contrasting Patterns of the Bacterial Communities in Melting Ponds and Periglacial Rivers of the Zhuxi glacier in the Tibet Plateau
Source: Microorganisms. 2020 Apr 2;8(4):509. doi: 10.3390/microorganisms8040509 (PMC7232332; doi:10.3390/microorganisms8040509)
Supplement: Supplementary file 1 [file microorganisms-08-00509-s001.zip › Fig_S4.pdf]

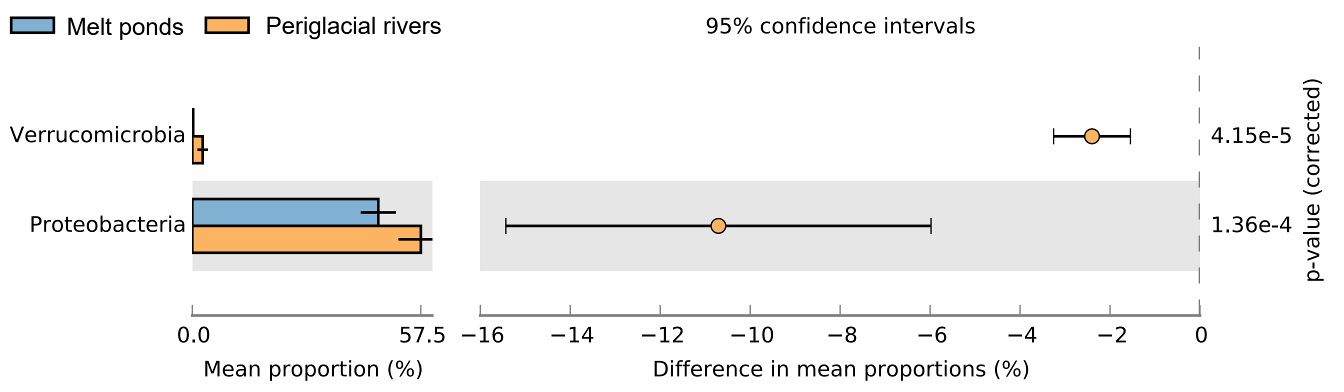

Melt ponds
Periglacial rivers
95% confidence intervals
Verrucomicrobia
Proteobacteria
4.15e-5
1.36e-4
p-value (corrected)
0.0
57.5
−16
−14
−12
−10
−8
−6
−4
−2
0
Mean proportion (%)
Difference in mean proportions (%)
