# Supplementary material for: Contrasting Patterns of the Bacterial Communities in Melting Ponds and Periglacial Rivers of the Zhuxi glacier in the Tibet Plateau
Source: Microorganisms. 2020 Apr 2;8(4):509. doi: 10.3390/microorganisms8040509 (PMC7232332; doi:10.3390/microorganisms8040509)
Supplement: Supplementary file 1 [file microorganisms-08-00509-s001.zip › Fig_S5.pdf]

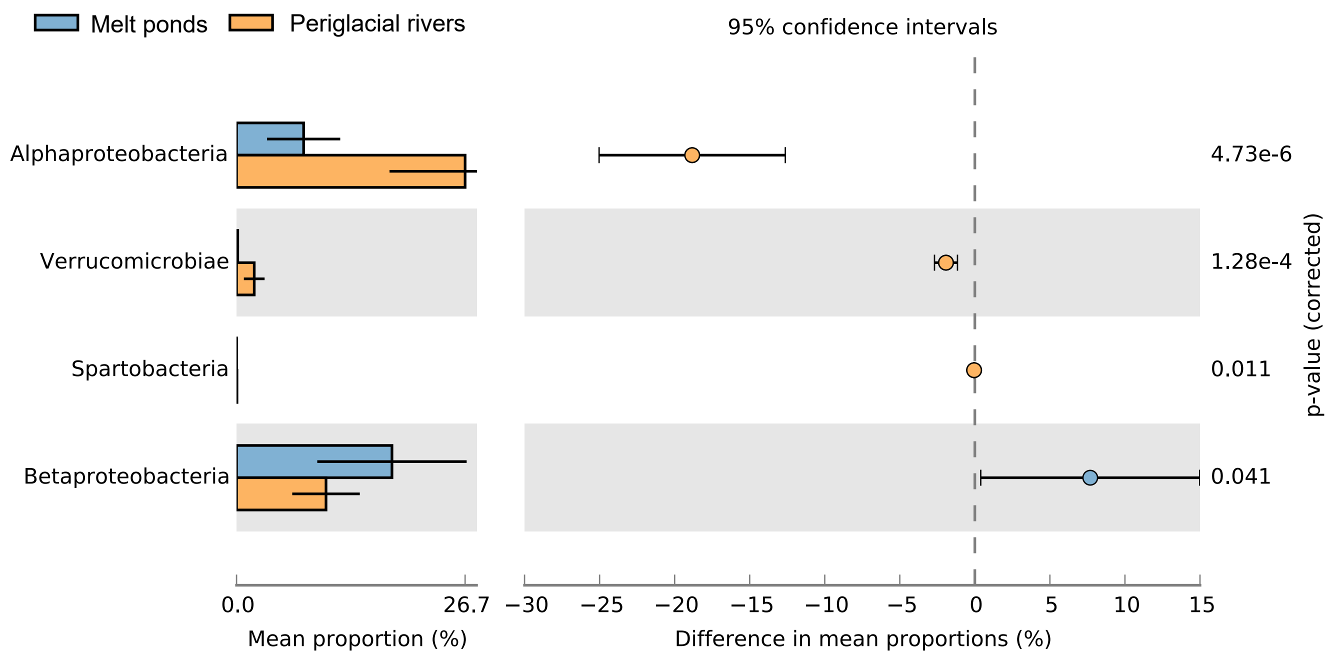

Melt ponds
Periglacial rivers
95% confidence intervals
Alphaproteobacteria
Verrucomicrobiae
Spartobacteria
Betaproteobacteria
4.73e-6
1.28e-4
0.011
0.041
p-value (corrected)
0.0
26.7
Mean proportion (%)
−30
−25
−20
−15
−10
−5
0
5
10
15
Difference in mean proportions (%)
