# Supplementary figures and images for: Contrasting Patterns of the Bacterial Communities in Melting Ponds and Periglacial Rivers of the Zhuxi glacier in the Tibet Plateau
Source: Microorganisms. 2020 Apr 2;8(4):509. doi: 10.3390/microorganisms8040509 (PMC7232332; doi:10.3390/microorganisms8040509)

Melt ponds      Periglacial rivers

95% confidence intervals

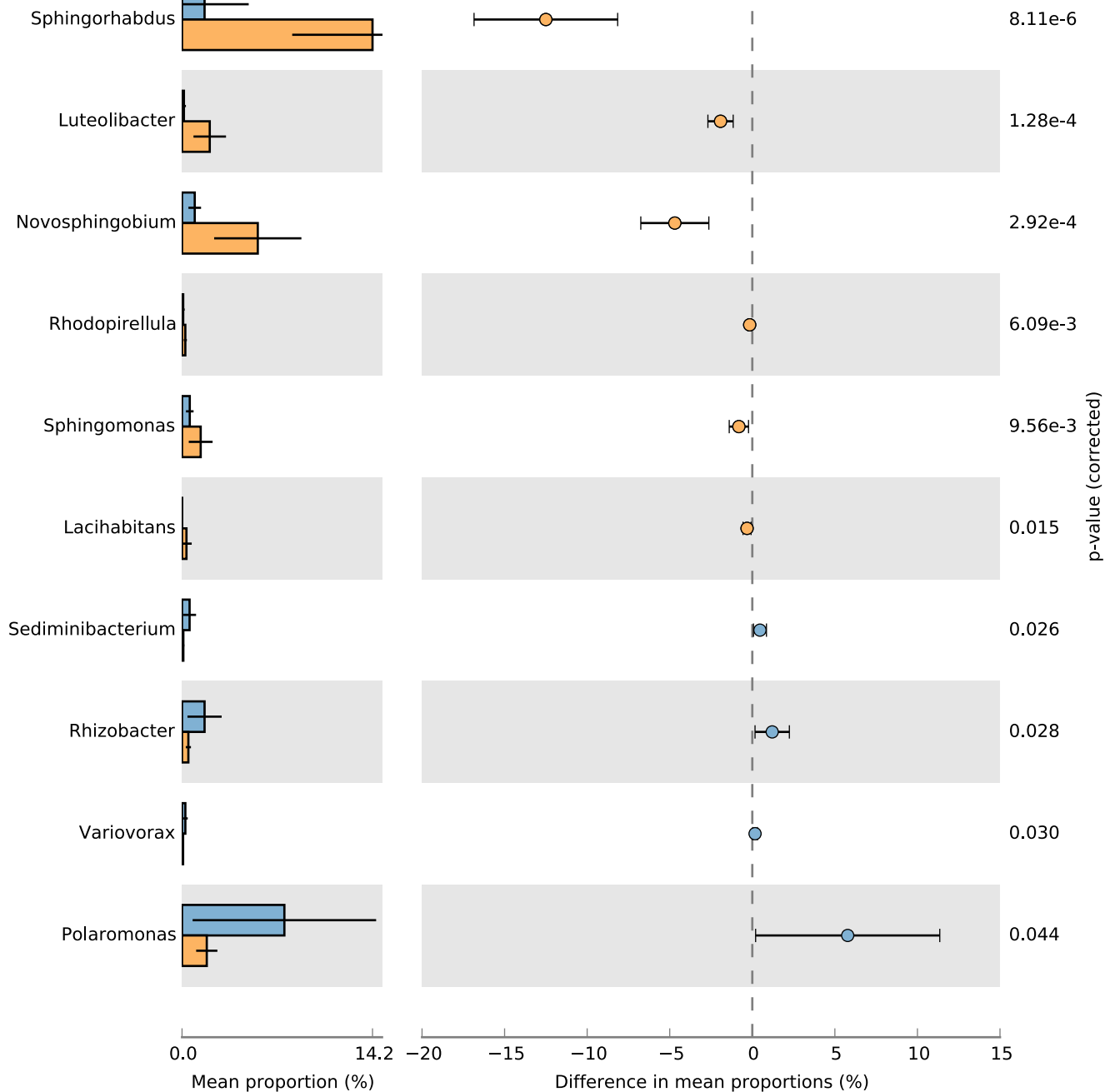

Supplement: Supplementary file 1 [file microorganisms-08-00509-s001.zip › Fig_S6.pdf]

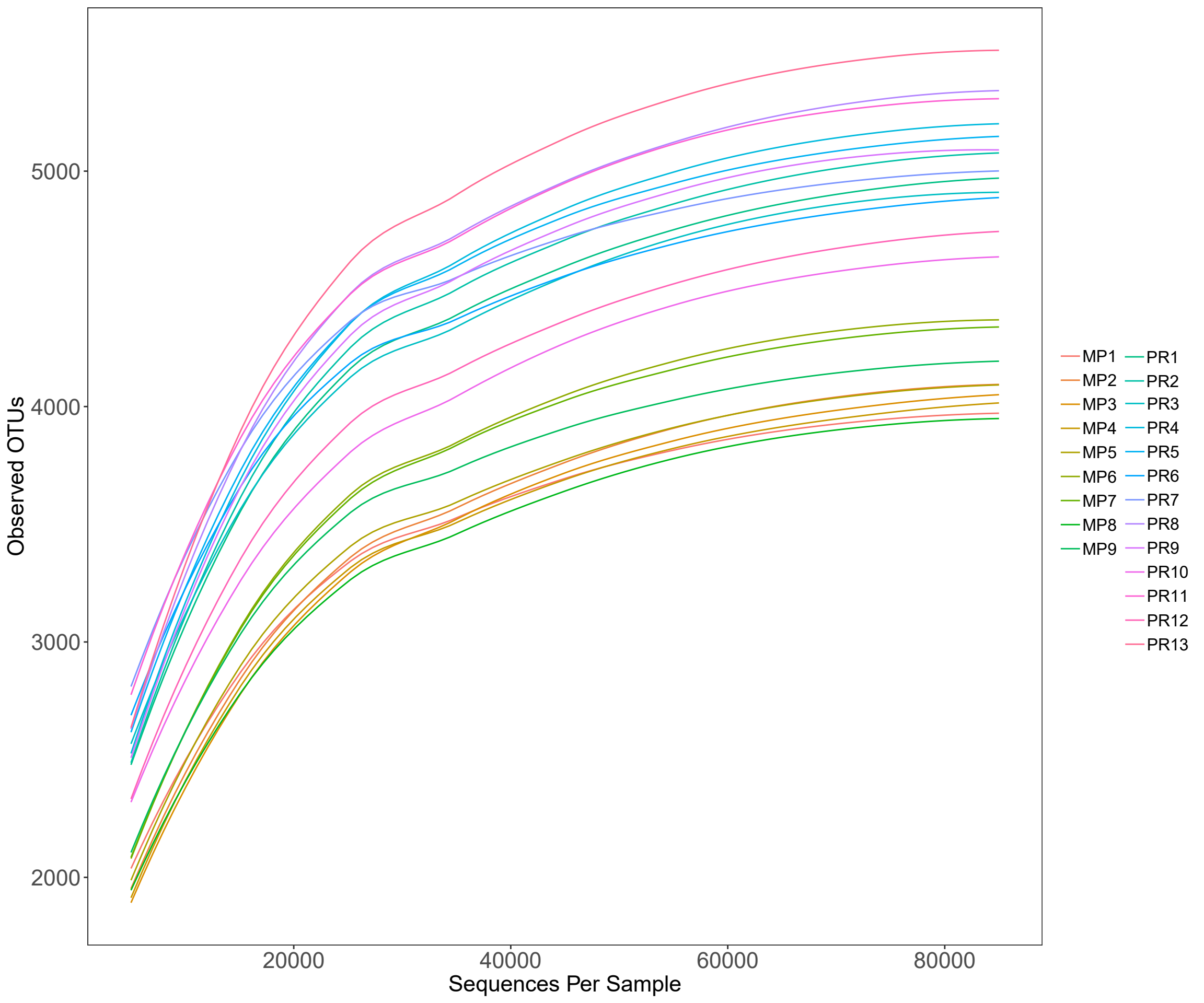

Supplement: Supplementary file 1 [file microorganisms-08-00509-s001.zip › Fig_S1.pdf]

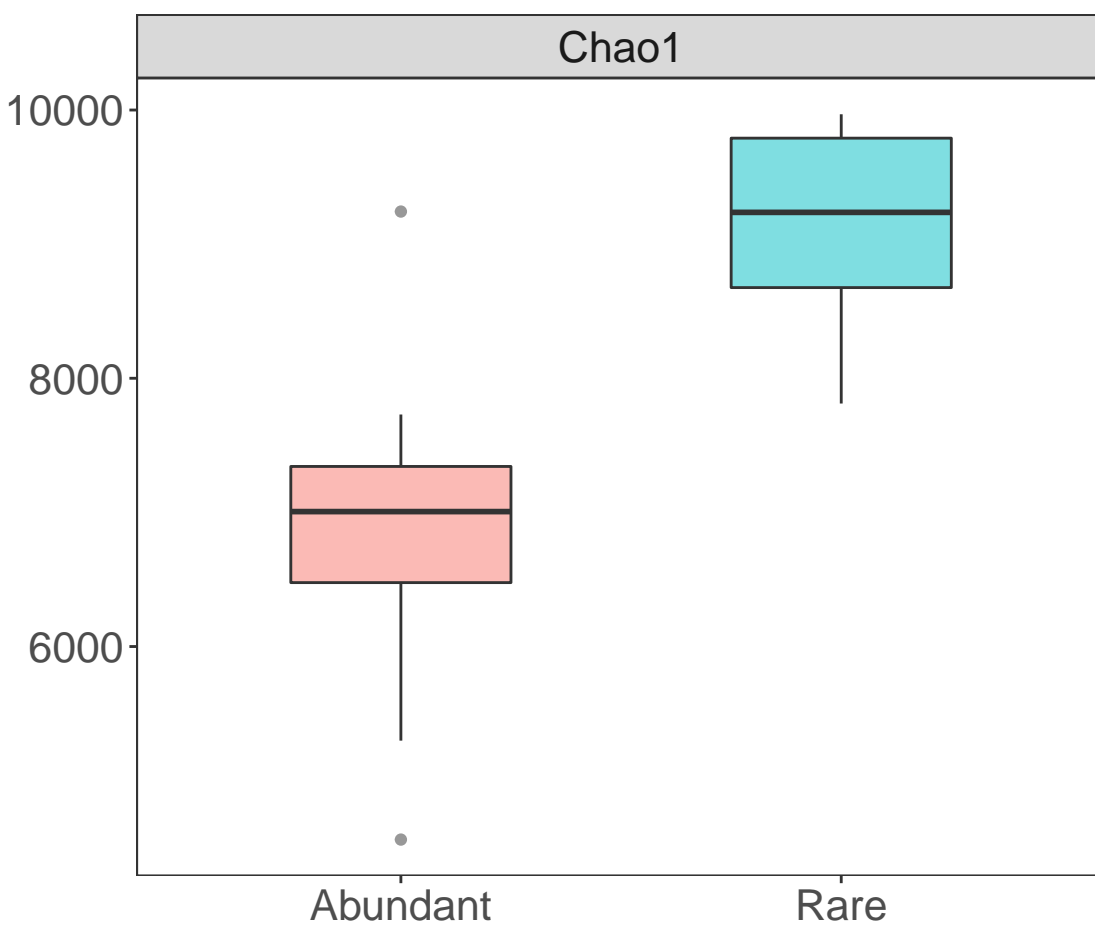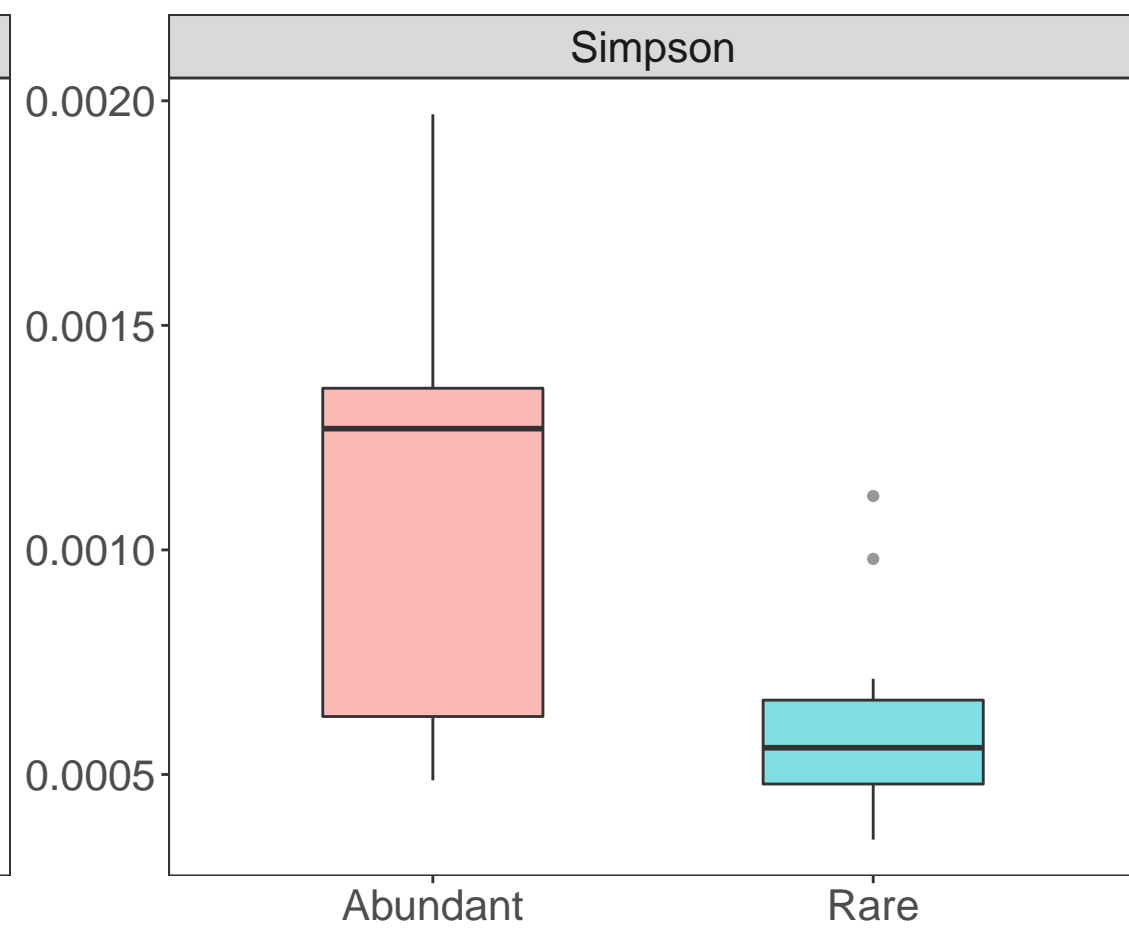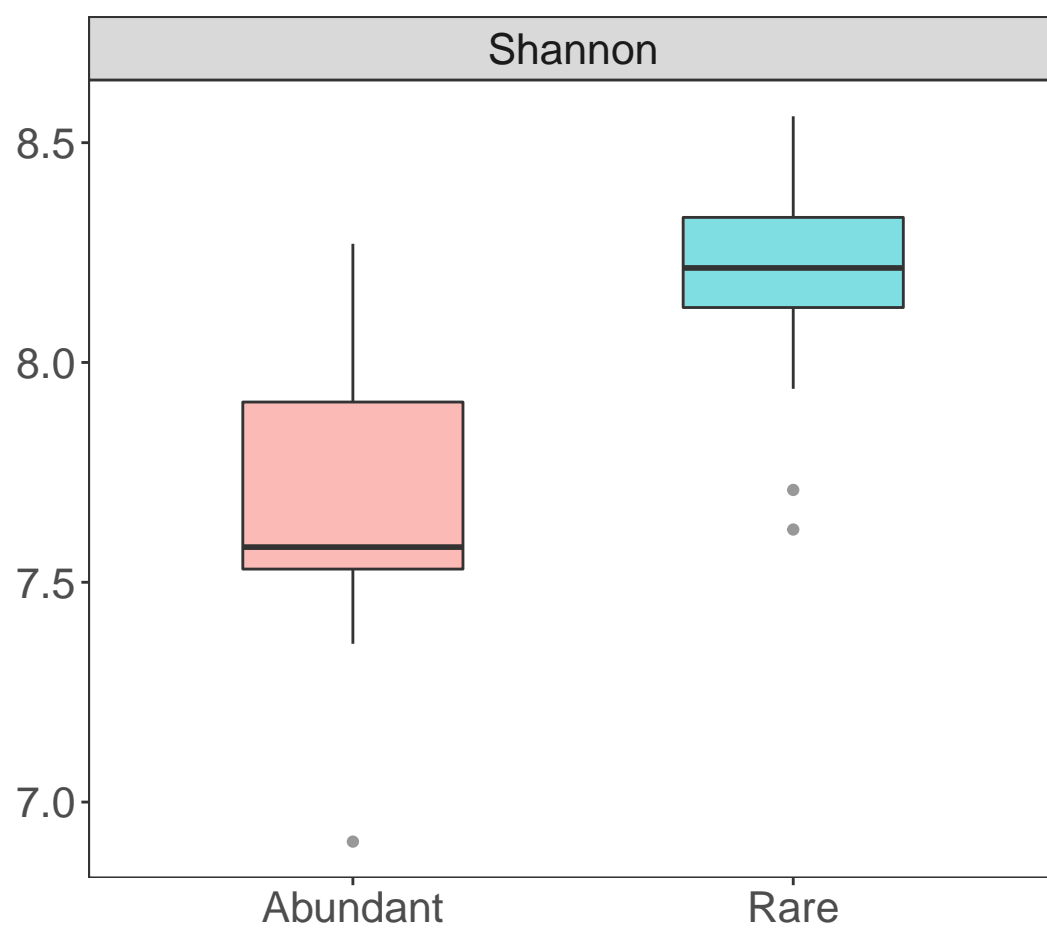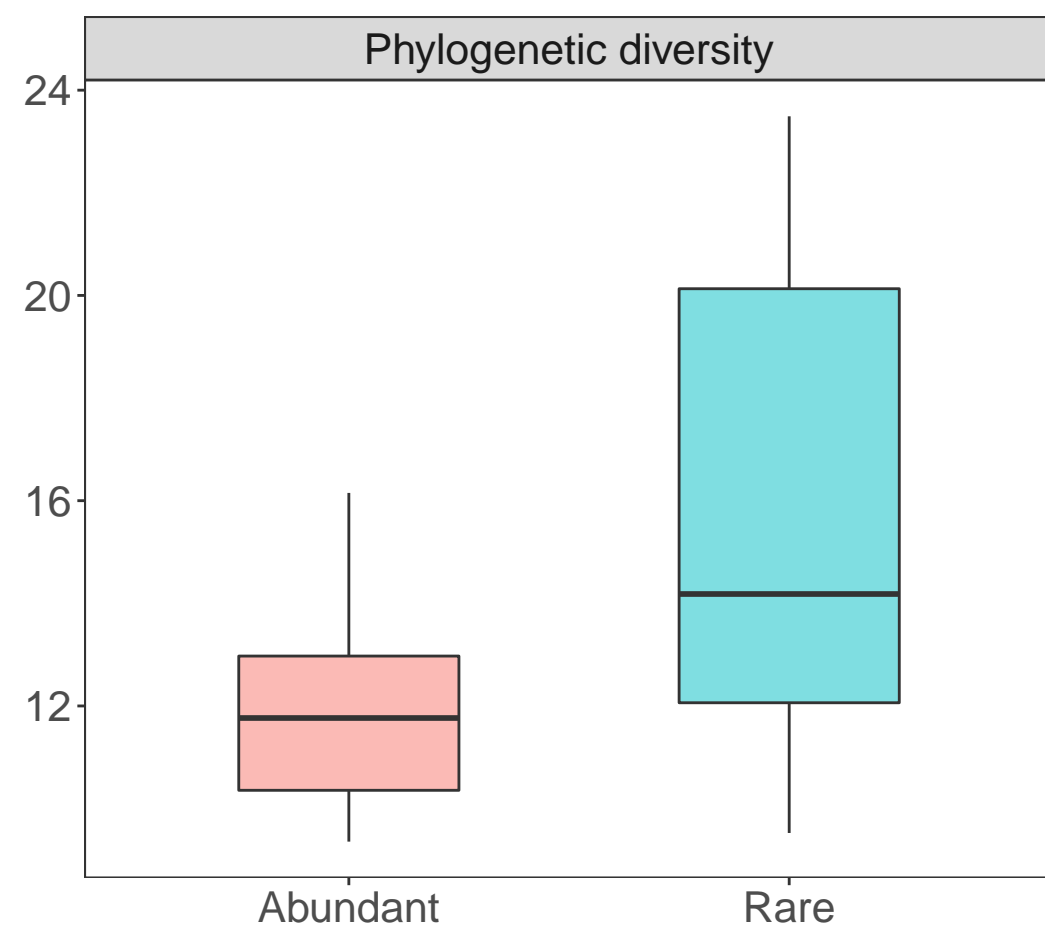

Supplement: Supplementary file 1 [file microorganisms-08-00509-s001.zip › Fig_S2.pdf]

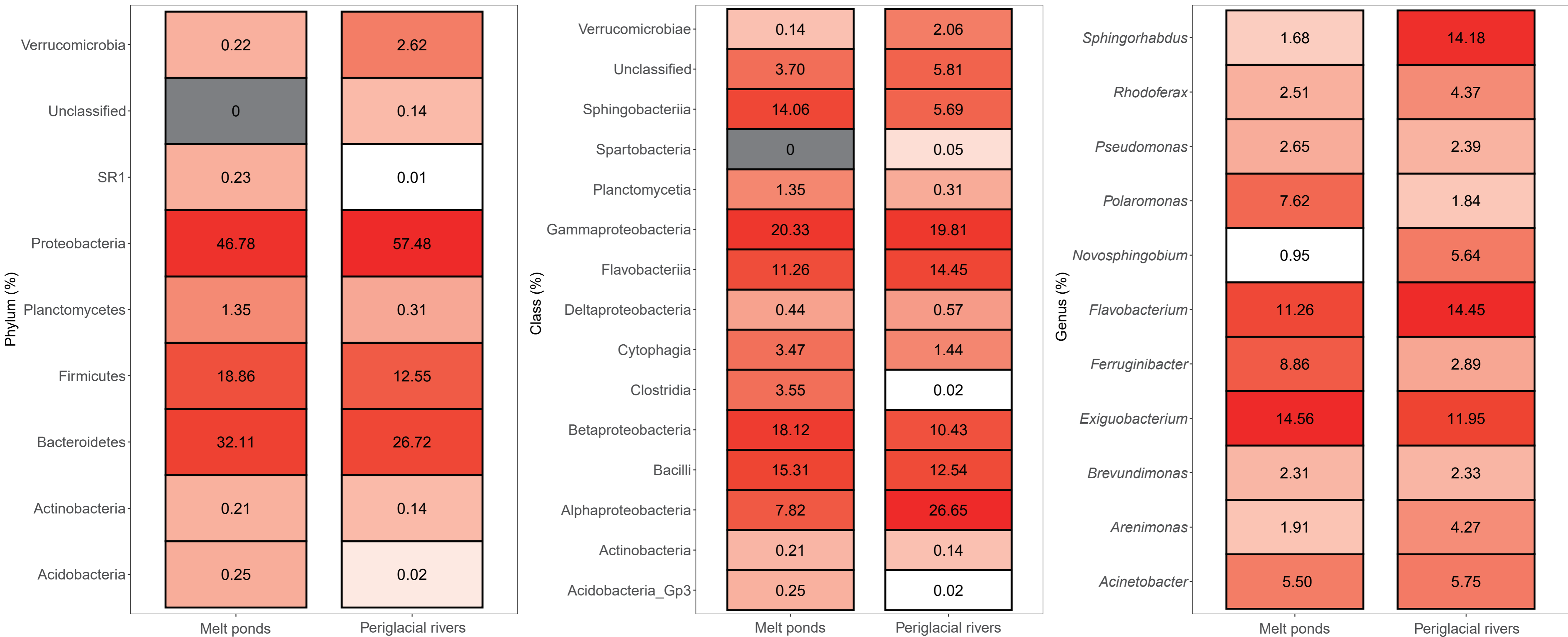

Supplement: Supplementary file 1 [file microorganisms-08-00509-s001.zip › Fig_S3.pdf]
